# Assessing Cue-Induced Brain Response as a Function of Abstinence Duration in Heroin-Dependent Individuals: An Event-Related fMRI Study

**DOI:** 10.1371/journal.pone.0062911

**Published:** 2013-05-07

**Authors:** Qiang Li, Yarong Wang, Yi Zhang, Wei Li, Jia Zhu, Ying Zheng, Jiajie Chen, Liyan Zhao, Zhenyu Zhou, Yijun Liu, Wei Wang, Jie Tian

**Affiliations:** 1 Department of Radiology, Tangdu Hospital, the Fourth Military Medical University, Xi’an, China; 2 Life Sciences Research Center, School of Life Sciences and Technology, Xidian University, Xi’an, China; 3 National Institute on Drug Dependence, Peking University, Beijing, China; 4 Department of Psychiatry, McKnight Brain Institute, University of Florida, Gainesville, Florida, United States of America; 5 College of Engineering, Peking University, Beijing, China; 6 Institute of Automation, Chinese Academy of Sciences, Beijing, China; Yale University, United States of America

## Abstract

The brain activity induced by heroin-related cues may play a role in the maintenance of heroin dependence. Whether the reinforcement or processing biases construct an everlasting feature of heroin addiction remains to be resolved. We used an event-related fMRI paradigm to measure brain activation in response to heroin cue-related pictures versus neutral pictures as the control condition in heroin-dependent patients undergoing short-term and long-term abstinence. The self-reported craving scores were significantly increased after cue exposure in the short-term abstinent patients (*t* = 3.000, *P = *0.008), but no increase was found in the long-term abstinent patients (*t* = 1.510, *P = *0.149). However, no significant differences in cue-induced craving changes were found between the two groups (*t* = 1.193, *P = *0.850). Comparing between the long-term abstinence and short-term abstinence groups, significant decreases in brain activation were detected in the bilateral anterior cingulated cortex, left medial prefrontal cortex, caudate, middle occipital gyrus, inferior parietal lobule and right precuneus. Among all of the heroin dependent patients, the abstinence duration was negatively correlated with brain activation in the left medial prefrontal cortex and left inferior parietal lobule. These findings suggest that long-term abstinence may be useful for heroin-dependent patients to diminish their saliency value of heroin-related cues and possibly lower the relapse vulnerability to some extent.

## Introduction

Heroin dependence is a complex disorder of the brain, involving both affective and cognitive processes, characterized by a compulsive drive to take drugs despite serious negative consequences [1]. The measure of inpatient abstinence is generally used in order to reduce the probability of relapse after detoxification. An existing problem with heroin dependence is that most heroin-dependent individuals will often relapse after certain periods of abstinence [2], especially after a short period of abstinence. It was demonstrated that relapse was highly associated with shorter treatment duration for drug addicted individuals [3]. Whether long-term abstinence has a positive effect on brain function of heroin-dependent individuals is an important issue which is not very clear. Investigation of heroin-related cues induced craving and brain response may be a key to solving this problem.

Emerging neuroimaging studies have shown an abnormal functional organization of brains in addictive populations, in which there is enhanced salience of drug-related cues but weakened strength of cognitive control [4]–[6]. In chronic heroin abusers, the literature of cue-reactivity fMRI demonstrates that heroin-related cues activate the ventral striatum (nucleus accumbens and caudate), putamen, anterior cingulate cortex (ACC), orbitofrontal cortex (OFC), dorsolateral prefrontal cortex (DLPFC), medial prefrontal cortex (MPFC), amygdala, posterior cingulate cortex (PCC), insula and thalamus, as well as increased craving [7]–[13]. However, whether the heroin-related cues induced response would change depending on abstinence status remains unclear. Exploring the alteration of the cue-induced response in the brain may provide a new perspective in understanding the effect of abstinence.

Recently, a study by Lou [14] demonstrated that long-term abstinence could decrease the heroin-related cues induced brain response mainly involved in posterior parts of the brain, such as the occipital cortex, temporal cortex, parietal cortex and subcortical regions. However, no change was found in the prefrontal regions. The prefrontal regions, such as the OFC, DLPFC and MPFC, play an important role in cognitive control [5], [15] and impairments in these regions may be the key reason for relapse after periods of abstinence. Therefore, it is crucial to know whether or not the heroin-cue induced abnormally greater responses in the prefrontal regions would decrease during long-term abstinence. Functional changes in the prefrontal cortex may be essential for heroin dependent patients to relapse or not during a long term of abstinence. From this perspective, it is important to determine if there would be any functional recovery in the prefrontal regions as the result of long-term abstinence.

In the present study, we used an event-related fMRI paradigm and cross-sectional design to investigate a cohort of abstinent heroin patients who varied in the duration of their abstinence. Comparisons between the short-term abstinent (SA) group and long-term abstinent (LA) group allowed us to determine whether the heroin-cues-induced responses were correlated with abstinence duration. We hypothesized that long-term abstinence would lead to decreases in the cue-induced craving as well as brain responses, especially in the prefrontal regions.

## Materials and Methods

### Ethics Statement

This study was carried out at Tangdu Hospital and approved by the Ethics Committee of Tangdu Hospital. All subjects signed an informed consent prior to study participation.

### Subjects

Thirty-seven abstinent heroin-dependent patients were recruited from the Drug Rehabilitation Center in Lantian, Xi’an. After undergoing methadone-assisted heroin detoxification, they were under treatment including psychological rehabilitation and vocational training programs. These patients were drug-free while staying in the Drug Rehabilitation Center prior to the current fMRI study. The patients’ general cognitive function was assessed by an experienced doctor in the Drug Rehabilitation Center through a face-to-face interview. The Beck Depression Inventory (BDI) and Hamilton Anxiety Scale (HAMA) were used to evaluate the severity of depression and anxiety symptoms respectively. They were divided into the SA group (n = 19) and LA group (n = 18). The two groups were demographically matched (Table 1). All of the abstinent patients met the criteria for heroin dependence for at least 1 year, as diagnosed by the Structured Clinical Interview for DSM-IV. To make sure the participants were in the detoxification phase, all of the abstinent patients tested negative for the presence of morphine in the urinalysis. The inclusion criteria also included (1) aged 18–50 years old; (2) right-handed; and (3) no somatic symptoms of withdrawal and negative naloxone tests. The exclusion criteria included: (1) current or past psychiatric illness other than heroin dependence; (2) current or past major medical illnesses or current use of prescription medications; (3) dependence on other psychoactive substances (except nicotine); (4) having a history of head injury or neurological illness; and (5) claustrophobia or other medical conditions that would preclude the patient from lying in the MRI scanner for approximately 40 minutes.

**Table 1:** Demographic and clinical characteristics of the participants.

Characteristics	PA N = 18	SA N = 19	*t*-value	*P*-value
**Age**	34.6±6.8	32.2±6.5	1.095	0.281
***Range***	22–45	23–44	–	–
**Years of education**	8.8±2.3	9.7±2.2	–1.320	0.195
***Range***	6–15	6–12	–	–
**Duration of heroin use (months)**	96.3±69.5	80.5±54.4	0.770	0.446
***Range***	22–245	12–229	–	–
**Average heroin dose (g/day)**	0.8±0.4	1.1±1.3	0.864	0.393
***Range***	0.2–2	0.1–3.5	–	–
**Total heroin dose (g)**	2343.2±2229.8	2385.8±2701.0	0.052	0.959
***Range***	250–7350	108–8400	–	–
**Abstinence (days)**	193.3±42.7	23.6±17.6	–	–
***Range***	150–300	7–72	–	–
***BDI***	13.6±7.6	9.9±6.8	1.568	0.127
***Range***	1–30	0–23	–	–
***HAMA***	7.9±6.9	7.9±7.6	–0.024	0.981
***Range***	0–23	0–30	–	–

### Experimental Paradigm

We employed a previously established event-related design [7] in the cue-induced craving task. All subjects participated in a 490-s run during which they were exposed to 48 pictures (24 heroin pictures and 24 neutral pictures). The task began with 10 seconds of a dummy scan followed by the first stimulus picture. The task consisted of 48 trials and each trial included a 2-s picture (heroin-related or neutral picture) and an inter-stimulus interval (ISI) during which a crosshair was displayed. The ISI ranged from 4 to 12 s. Trials were presented in a pseudo-randomized order. Stimulus presentations were delivered by using the E-Prime software package (Psychology Software Tools, Inc., Pittsburgh, PA, USA). Participants were placed in the scanner in a supine position, using a foam head holder to reduce motion. Earplugs were used to lessen scanner noise. No use of cigarettes, alcohol, tea, caffeine and any other drug or medicine was allowed 4 h prior to the time of the MRI scan. Patients were given a ‘talkdown’ to decrease heroin craving or subjective withdrawal symptoms after the fMRI scan, which may have been elicited by the heroin-related cues.

### Data Acquisition

MRI was performed on a 3.0 T GE MRI system (General Electric, Milwaukee, Wisconsin) using an 8-channel phase array coil. Before the fMRI scan, high-resolution Fast Spoiled Gradient Recalled T1-weighted images were collected for spatial normalization [repetition time (TR) = 3.0 ms, echo time (TE) = 7.8 ms, matrix = 256×256, field of view (FOV) = 256×256 mm^2^, slice thickness = 1 mm, and spatial resolution = 1×1×1 mm^3^]. The brain structural data were checked by an experienced radiologist to assure that there was no structural abnormality. The functional images were collected using gradient Echo Planar Imaging (EPI) (TR = 2 s, TE = 30 ms, matrix = 64×64, FOV = 256×256 mm^2^, slice thickness = 4 mm, gap = 0 mm and flip angle = 90 degrees) with in-plane resolution of 4×4 mm^2^. For each subject, 240 echo-planar volumes were collected respectively during the cue-reactivity run.

Heroin craving was assessed by a 0–10 visual analog scale (VAS) [7] from each heroin-dependent participant before and after the cue-reactivity run. The question “To what extent do you feel the urge to use heroin?” was used to get heroin craving ratings (0 for the least craving and 10 for the strongest craving).

### Data Processing

The fMRI Data processing was conducted using Statistical Parametric Mapping (SPM) 8 software (http://www.fil.ion.ucl.ac.uk/spm). All of the fMRI images acquired were slice time corrected with reference to the first slice, corrected for motion artifacts by realignment to the last volume, and spatially registered to the high-resolution Fast Spoiled Gradient Recalled T1-weighted images and then normalized to a standard SPM T1 template. The images were resampled to 3×3×3 mm^3^ and smoothed with an isotropic 4-mm full-width half-maximum Gaussian kernel. Subjects with head motion more than 1.5 mm maximum displacement in any direction of the *x*, *y* and *z* planes or 1.5 degrees of any angular motion throughout the course of the scan were excluded. The first-level analysis was conducted by modeling the drug and neutral cues condition as explanatory variables within the context of a general linear model in a voxel-wise manner with SPM8. The contrast of drug versus neutral was used to assess differences in brain responses to heroin cues. In the second-level analysis, we used individual contrast maps (heroin versus neutral cues) of all of the participants in each group to determine the group differences. A two-sample *t*-test was performed to determine activation volume differences in brain regions between groups. For all of the whole-brain contrasts, a significance threshold corresponding to *P*<0.001 was applied in combination with a minimum cluster size threshold of 6 voxels to correct for false positives (*P*<0.05) based on 10,000 Monte Carlo simulations [16]. All coordinates reported were in the Montreal Neurological Institute space.

For all of the heroin-dependent patients, voxel-wise correlations were performed to assess the relationship between abstinence duration and brain activation amplitude between viewing heroin-related and neutral pictures. The heroin use history (duration of heroin use, heroin dose per day and total heroin dose) was used as a covariant. The correlations between changes in craving and brain activation amplitude were also performed. Significance was set at *P*<0.05 (corrected with Monte Carlo simulation).

## Results

### Craving Measures

For the SA group, the subjective craving score before and after cue exposure and change in craving was 2.5±1.7, 3.8±2.7 and 1.2±1.8 respectively. For the LA group, the subjective craving score before and after cue exposure and change in craving was 1.5±2.0, 2.5±2.4 and 1.1±2.9 respectively. The paired sample *t*-test indicated that subjective craving scores after cue exposure were significantly higher than before cue exposure for the SA group (*t* = 3.000, *P = *0.008), but not for the LA group (*t* = 1.510, *P = *0.149). No significant difference in the craving score before, after cue exposure (*t* = 1.659, *P = *0.108; *t* = 1.386, *P = *0.173) and craving change (*t* = 1.193, *P = *0.850) was found between the two groups.

### The within-group Differences

**Figure 1:**
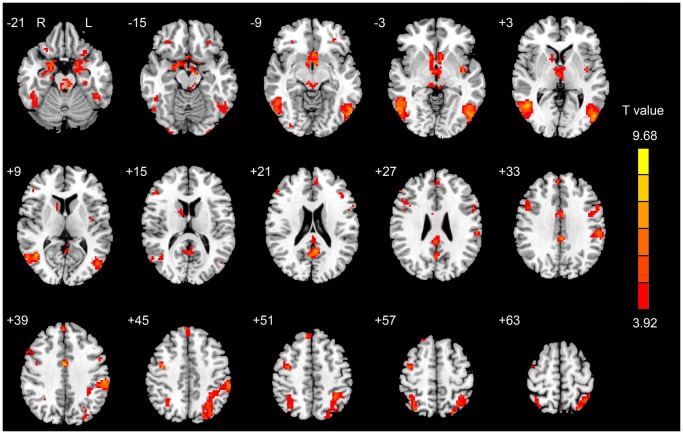
The activated regions relating to the “Heroin-Neutral” contrast for the SA group (*P*<0.05, corrected for Monte Carlo simulations).

**Figure 2:**
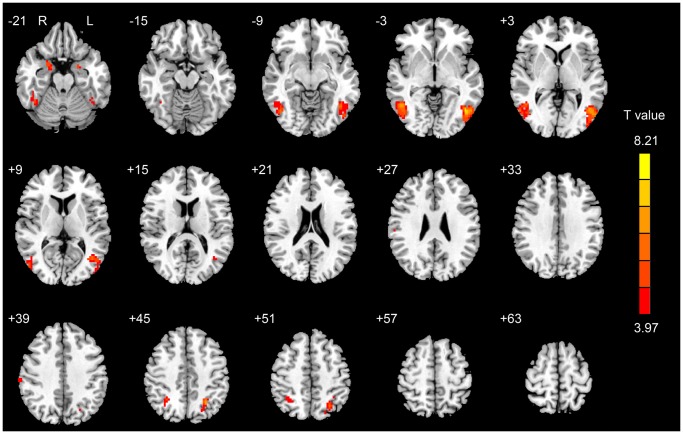
The activated regions relating to the “Heroin-Neutral” contrast for the LA group (*P*<0.05, corrected for Monte Carlo simulations).

In the SA group, the significantly activated brain regions relating to the “Heroin-Neutral” contrast included the bilateral nucleus accumbens (NAc), caudate, DLPFC, OFC, MPFC, superior frontal gyrus (SFG), precentral gyrus (PrG), amygdala, pons, midbrain, middle cingulate cortex (MCC), fusiform, PCC, precuneus, thalamus, middle occipital gyrus (MOG), middle temporal gyrus (MTG), inferior temporal gyrus (ITG), inferior parietal lobule (IPL), left ACC, hippocampus, insula, inferior occipital gyrus (IOG), angular gyrus and cerebellum (Figure 1 and Table S1). In the LA group, the significantly activated brain regions included the left amygdala, inferior occipital gyrus (IOG), superior parietal lobule (SPL) and right parahippocampal gyrus, angular gyrus and bilateral fusiform, IPL, ITG, MTG, MOG and cerebellum (Table 2 and Figure 2).

**Table 2:** Activated brain regions for the LA group in response to heroin-related vs. neutral cues.

Brain regions		Brodmann’s area	Peak location				Peak *t*-score	Voxel number
			x	y		z		
Amygdala	L	–	−21		−3	−24	5.67	7
Angular Gyrus	R	7	30		−56	48	4.50	13
Cerebellum	L	–	−40		−47	−27	4.36	10
	R	–	44		−53	−27	6.46	13
Fusiform	L	37	−42		−49	−24	4.88	10
	R	37	42		−51	−24	5.45	18
IOG	L	37	−45		−66	−3	8.21	54
IPL	L	7	−27		−57	45	6.75	14
	R	7	30		−54	48	5.21	15
ITG	L	37	−51		−58	−7	3.80	40
	R	19	47		−69	−6	5.48	34
MTG	L	37	−51		−66	0	6.86	54
	R	37	51		−57	0	7.42	109
MOG	L	37	−48		−66	0	7.20	62
	R	37	44		−69	7	4.22	15
Parahippocampal gyrus	R	28	24		3	−24	6.52	16
Supramarginal gyrus	R	2	66		−27	30	4.73	12
SPL	L	7	−27		−59	51	5.00	47

### The between-group Differences

**Figure 3:**
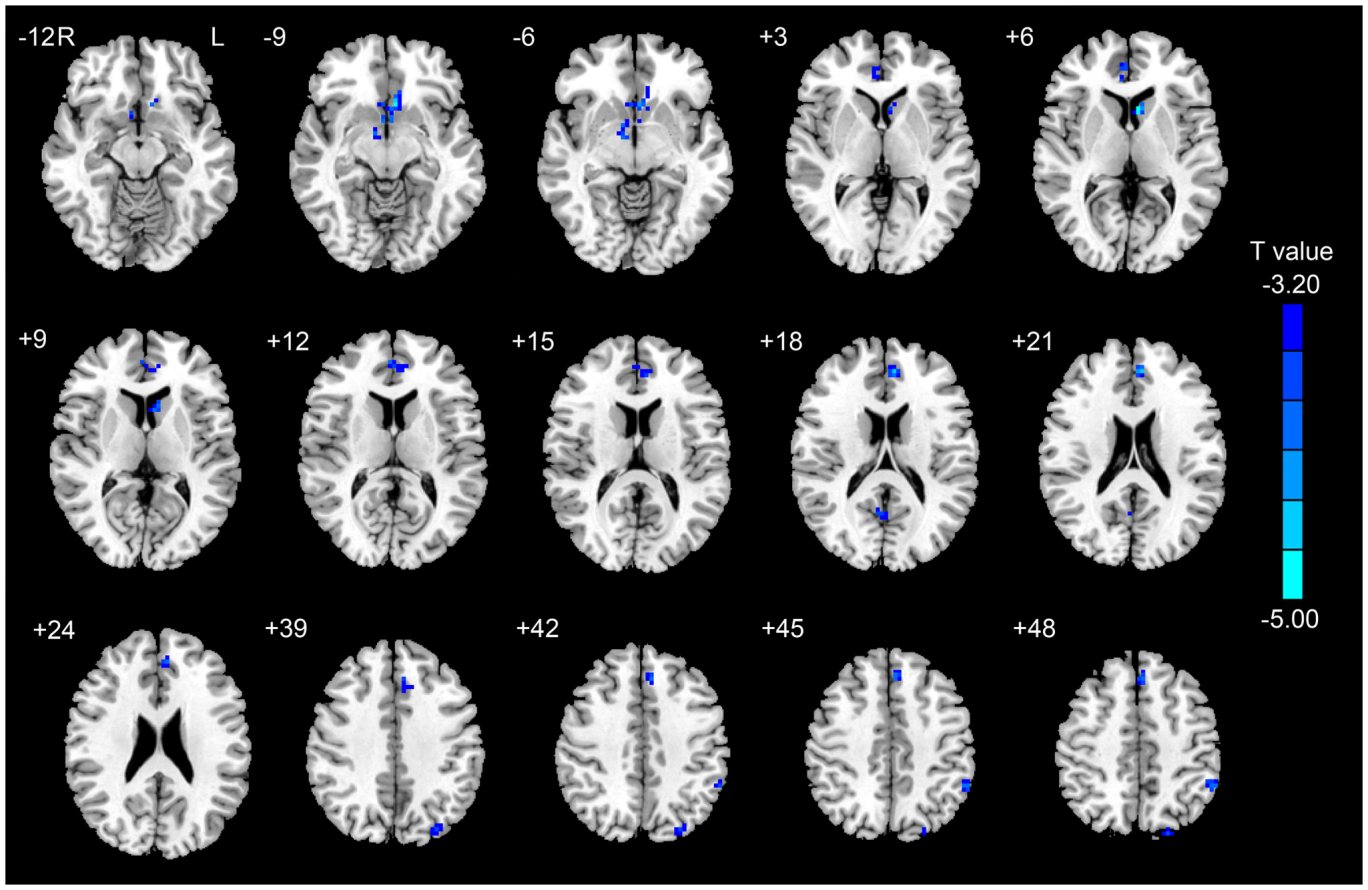
The differences relating to the “Heroin-Neutral” contrast between the LA and SA groups (*P*<0.05, corrected for Monte Carlo simulations).

Compared to the SA group, the LA group had significantly lower activation in the following regions: bilateral ACC, left MPFC, caudate, MOG, IPL and right precuneus. The LA group did not show greater activation in any brain regions relative to the SA group in response to heroin-related cues (Table 3 and Figure 3).

**Table 3:** Activated brain regions for the LA group in contrast to the SA group in response to heroin-related vs. neutral cues.

Brain regions		Brodmann’s area	Peak location				Peak *t*-score	voxel number
			x	y	z			
ACC	L	32	−6	46		16	−3.48	19
	R	32	5	46		7	−3.65	13
Caudate	L	–	−6	15		6	−5.00	13
Cerebellum	L	–	−9	−72		−42	−3.90	13
IPL	L	40	−57	−48		45	−4.01	33
MPFC	L	32	−6	45		21	−4.50	20
MOG	L	19	−28	−84		40	−3.43	11
Precuneus	R	30	6	−56		18	−3.76	10

### Correlation Results

SPM voxel-wise correlation between abstinence duration and signal change in response to heroin versus neutral cues demonstrated a significant negative correlation in a cluster region (13 voxels) located in the left MPFC, which was centered at MNI x, y, z coordinates of −6, 45, 21 (peak *r* = −0.60) (*P*<0.05, corrected with Monte Carlo simulation). We also found another significant negative correlation in a cluster region (60 voxels) located in the left IPL; centered at MNI x, y, z coordinates of −48, −57, 54 (peak *r* = −0.57) (*P*<0.05, corrected with Monte Carlo simulation). (Figure S1) There were no significant correlations between changes in craving and brain responses to heroin versus neutral cues.

## Discussion

Employing the event-related fMRI paradigm, we found that the LA group demonstrated significantly decreased activity to heroin-related cues than the SA group in the cognitive control related regions (bilateral ACC and left MPFC), reward related region (left caudate), and visuospatial attention regions (right precuneus and left IPL). Significant craving increase was induced by heroin-related cues in the SA group but no change in craving was found in the LA group.

Our results demonstrated that in the SA group, the heroin-related cues significantly induced the brain response in the mesolimbic regions including the ACC, caudate, NAc, amygdala, hippocampus, PCC and the prefrontal regions including the DLPFC, OFC, MPFC and visuospatial attention regions including the precuneus, IPL, ITG, MTG, IOG, and MOG. These results were consistent with previous studies on the drug-cue-induced brain response [7], [8], [17], [18]. Our findings reflected that when the heroin-dependent patients just finished detoxification, they still had strong brain activation and an increased craving level for heroin use in response to the heroin-related cues. However, the data indicated that the LA group had a significantly decreased brain response to the heroin-related stimuli and unchanged craving level, although craving changes in the two groups were similar. Therefore, we postulated that long-term abstinence may be useful to help heroin-dependent patients reduce the heroin-related saliency value of conditioned cues and benefit heroin addiction therapy.

The ACC and MPFC were found to cover the majority of the differential voxels between the LA and SA groups. Activation of the ACC has been frequently identified during drug craving [19]. The ACC is involved in drug-seeking behavior [20], partly via its roles in reward-related decision-making and cognitive control [21], [22]. The MPFC is implicated in decision-making, emotional information processing, and goal-directed action planning [23], [24]. The decreased cue-induced activity of the ACC and MPFC in the LA group and the negative correlation between MPFC activity and abstinence duration among all the heroin dependent patients might reflect the function of cognitive control recovered to some extent during long-term abstinence. These results are consistent with what Goldstein and Volkow proposed recently that long-term abstinence attenuates cue-induced responses in the prefontal cortex [25].

For the drug-dependent patients, the enhanced activity in the caudate in response to drug-related cues indicated increased reward-based cognitive processes in the presence of the cues [26]. The significantly decreased caudate activity in the LA group compared with the SA group might reflect that long-term abstinence could attenuate the abnormally increased reward-based cognitive processes. It might also explain why the craving level of the LA group remained unchanged after the heroin-related-cues exposure.

The precuneus and IPL are involved in visuospatial attention [27]–[29]. Activity of the precuneus and IPL for drug-related cues compared with control cues has been revealed by several studies [9], [19]. The precuneus plays a role in motor imagery [30] and mental simulation [31] and the IPL seems to link perception and action [32]. The decreased activity in the right precuneus and left IPL together with the negative correlation between left IPL activity and abstinence duration among all the heroin dependent patients might reflect that the imagery and/or preparation of approach-related action to the heroin-related cues was attenuated with abstinence duration in abstinent heroin patients.

The results of the current study indicated that these changes in brain activity after long-term abstinence treatment may help patients avoid the dangerous addictive behavior that culminates in relapse and may be used as a reliable marker of treatment efficacy. Future studies should focus on these brain regions mentioned above to further the exploration of effective treatment. However, the fact that the LA group demonstrated decreased neuronal reactivity to heroin-related cues during abstinence did not necessarily warrant a decreased risk for relapse. Most importantly, stress can also increase drug craving and contribute to relapse in drug dependence besides drug cue exposure [33]. Nevertheless, our results demonstrated that long-term abstinence may be useful in attenuating the saliency value of heroin-related cues which is very important for relapse.

Preclinical studies in rodents [34], [35] and nonhuman primates [36] showed that longer abstinence may have incubation effects, which would result in an increased rate of relapse. Our results seemed contradictory to the preclinical studies. The discrepancy between clinical and preclinical data might be explained by the following reasons. Lu and Grimm [37] acknowledged that the incubation of drug craving is long-lasting, but not permanent. They found in cocaine-dependent rats that cocaine seeking induced by exposure to cocaine cues remained elevated for up to 3 months after withdrawal, but decreased after 6 months. However, the mean abstinence duration was less than 1 month in the SA group and more than 6 months in the LA group. Due to the limitation of our sample type and sample size, we couldn’t tell whether or not there were incubation effects in heroin-dependent patients. Therefore, many more systematic studies are needed to identify whether there would be incubation effects in heroin-dependent patients.

### Limitations

One limitation of the present study was that we didn’t follow the patients throughout their relapses because it was very difficult to do so. Our patients came from many distant provinces. When they finished the treatment in the Drug Rehabilitation Center, they went back to their hometown. It was not convenient to contact them in person. Additionally, considering the negative aspect of their addictive behavior, they were unwilling to tell us any further personal information. Another limitation was that although this study employed a between-subject cross-sectional design rather than a within-subject longitudinal design, the investigation of the difference in the brain response to heroin-related cues as a function of abstinence duration would identify neurobiological characteristics related to successful abstinence. The last limitation was that we did not record eye tracking nor did we require responses during cue exposure. However, following the scanning session, each participant was interviewed and they could all remember most of the stimuli pictures.

### Conclusions

Using an event-related fMRI paradigm, we found that there was decreased brain response in heroin-dependent patients during long-term abstinence but no change in their craving level when being exposed to heroin-related cues. These findings suggest that long-term abstinence may be useful for heroin-dependent patients to diminish their saliency value of heroin-related cues and possibly lower the relapse vulnerability to some extent.

## Supporting Information

Figure S1 **Correlation maps between signal changes in the left MPFC and IPL, which were significantly activated among heroin-dependent patients by heroin-related cues, and the abstinence duration (** ***P*** **<0.05, corrected for Monte Carlo simulations;** ***r*** **: correlation coefficient).**
(TIF)

Table S1 **Activated brain regions for the SA group in response to heroin-related vs. neutral cues.**
(DOC)
