# Supplementary figures and images for: Assessing Cue-Induced Brain Response as a Function of Abstinence Duration in Heroin-Dependent Individuals: An Event-Related fMRI Study
Source: PLoS One. 2013 May 7;8(5):e62911. doi: 10.1371/journal.pone.0062911 (PMC3646913; doi:10.1371/journal.pone.0062911)

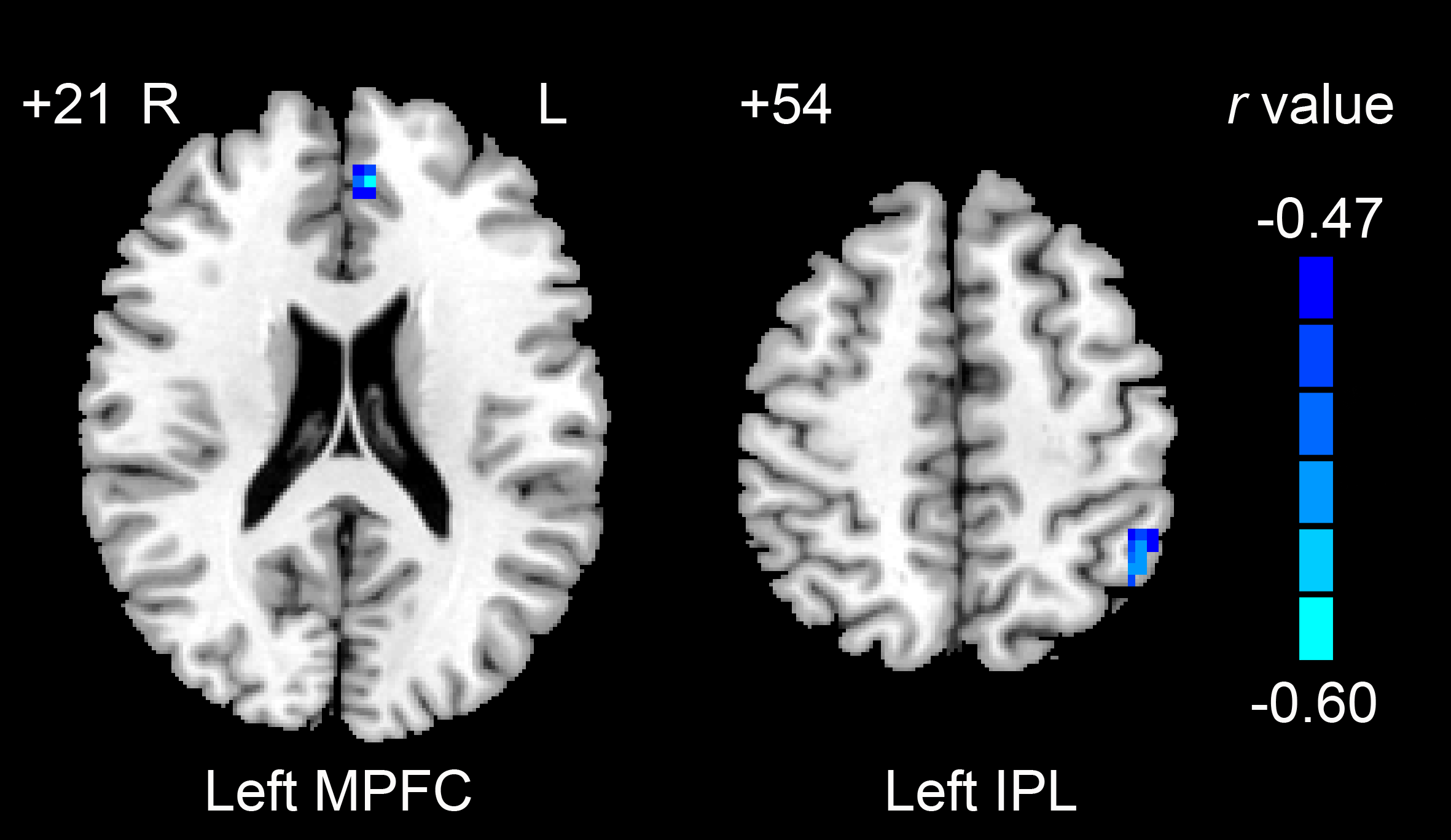

Supplement: Figure S1 — Correlation maps between signal changes in the left MPFC and IPL, which were significantly activated among heroin-dependent patients by heroin-related cues, and the abstinence duration ( P <0.05, corrected for Monte Carlo simulations; r : correlation coefficient). (TIF) [file pone.0062911.s001.tif]
