# Supplementary material for: Assessing Cue-Induced Brain Response as a Function of Abstinence Duration in Heroin-Dependent Individuals: An Event-Related fMRI Study
Source: PLoS One. 2013 May 7;8(5):e62911. doi: 10.1371/journal.pone.0062911 (PMC3646913; doi:10.1371/journal.pone.0062911)
Supplement: Table S1 — Activated brain regions for the SA group in response to heroin-related vs. neutral cues. (DOC) [file pone.0062911.s002.doc]

**Supporting Information Table S1. Activated brain regions for the SA group in response to heroin-related vs. neutral cues.**

| **Brain regions** | | | | **Brodmann’s area** | **Peak location** | | | | **Peak *t*-score** | **Voxel number** |
| --- | --- | --- | --- | --- | --- | --- | --- | --- | --- | --- |
|  |  |  |  |  | **x** | **y** | | **z** |  |  |
| ACC | | | L | 32 | -6 | | 46 | 19 | 4.30 | 10 |
| Amygdala | | | L | - | -20 | | 1 | -20 | 4.6 | 13 |
|  |  |  | R | - | 23 | | 2 | -18 | 3.92 | 24 |
| Angular gyrus | | | R | 7 | 33 | | -66 | 54 | 5.51 | 25 |
| Caudate | | | L | - | -9 | | 6 | 12 | 4.33 | 12 |
|  |  |  | R | - | 12 | | 9 | 12 | 4.98 | 45 |
| Cerebellum | | | R | - | 18 | | -75 | -48 | 4.00 | 10 |
| DLPFC | | IFG | L | 44 | -54 | | 9 | 33 | 4.74 | 12 |
|  |  |  | R | 44 | 48 | | 9 | 30 | 4.53 | 14 |
|  |  | MFG | R | 6 | 36 | | -9 | 48 | 7.23 | 21 |
| Fusiform | | | L | 37 | -40 | | -51 | -16 | 4.33 | 18 |
|  |  |  | R | 37 | 48 | | -45 | -24 | 4.68 | 14 |
| Hippocampus | | | L | - | -21 | | -6 | -24 | 6.65 | 18 |
|  |  |  | R | - | 21 | | -3 | -18 | 5.88 | 19 |
| ParaHippocampal | | | L | - | -20 | | -7 | -25 | 6.65 | 45 |
|  |  |  | R | - | 21 | | -3 | -24 | 6.85 | 50 |
| Insula | | | L | - | -39 | | -6 | 12 | 3.21 | 21 |
| IOG | | | L | 37 | -53 | | -72 | -6 | 9.68 | 71 |
| IPL | | | L | 2 | -57 | | -30 | 36 | 7.72 | 239 |
|  |  |  | R | 40 | 36 | | -54 | 57 | 4.37 | 21 |
| ITG | | | L | 37 | -53 | | -64 | -6 | 7.45 | 101 |
|  |  |  | R | 19,37 | 55 | | -63 | -3 | 6.93 | 175 |
| MCC | | | L | 23 | 0 | | -36 | 33 | 6.52 | 11 |
|  |  |  | R | 24 | 3 | | 0 | 36 | 6.40 | 25 |
| Midbrain | | | L | - | -3 | | -30 | -6 | 6.22 | 14 |
|  |  |  | R | - | 9 | | -27 | -6 | 5.55 | 13 |
| MOG | | | L | 37 | -53 | | -66 | -2 | 5.50 | 78 |
|  |  |  | R | 18,19,37 | 49 | | -75 | 1 | 5.10 | 17 |
| MPFC | | | L | 32 | -3 | | 49 | 24 | 4.89 | 25 |
|  |  |  | R | 9 | 4 | | 48 | 36 | 5.00 | 29 |
| MTG | | | L | 37 | -47 | | -60 | -1 | 5.02 | 86 |
|  |  |  | R | 37 | 48 | | -57 | 3 | 8.30 | 195 |
| NAc | | | L | - | -6 | | 15 | -6 | 4.95 | 20 |
|  |  |  | R | - | 7 | | 12 | -7 | 4.37 | 17 |
| OFC | MFG | | L | 11, 47 | -28 | | 39 | -12 | 5.18 | 43 |
|  |  |  | R | 11,47 | 28 | | 37 | -11 | 4.00 | 27 |
| PCC | | | L | 30 | -4 | | -48 | 20 | 4.24 | 37 |
| Pons | | | L | - | -3 | | -30 | -21 | 6.65 | 14 |
| Precuneus | | | L | 23 | -3 | | -57 | 21 | 7.41 | 58 |
|  |  |  | R | 23 | 4 | | -57 | 18 | 5.29 | 18 |
| PrG | | | L | 6 | -48 | | 0 | 33 | 4.86 | 44 |
|  |  |  | R | 6 | 42 | | -3 | 45 | 6.23 | 15 |
| SFG | | | R | 8,9 | 3 | | 51 | 39 | 5.42 | 18 |
| Thalamus | | | L | - | -6 | | -9 | 1 | 4.50 | 13 |
|  |  |  | R | - | 4 | | -15 | 1 | 4.37 | 14 |
